# Whole blood transcriptomics reveals granulocyte colony‐stimulating factor as a mediator of cardiopulmonary bypass‐induced systemic inflammatory response syndrome

**DOI:** 10.1002/cti2.1490

**Published:** 2024-02-19

**Authors:** Katherine R Martin, Cristina Gamell, Tsin Yee Tai, Roberto Bonelli, Jacinta Hansen, James Tatoulis, Monther Alhamdoosh, Nicholas Wilson, Ian Wicks

**Affiliations:** ^1^ WEHI Parkville VIC Australia; ^2^ Department of Medical Biology University of Melbourne Parkville VIC Australia; ^3^ CSL Innovation, Bio21 Institute Parkville VIC Australia; ^4^ Cardiothoracic Surgery Royal Melbourne Hospital Parkville VIC Australia; ^5^ Department of Surgery University of Melbourne Parkville VIC Australia; ^6^ Department of Rheumatology Royal Melbourne Hospital Parkville VIC Australia

**Keywords:** cardiopulmonary bypass, cell‐free DNA, cytokines, granulocyte colony‐stimulating factor, systemic inflammatory response syndrome, whole blood transcriptomics

## Abstract

**Objectives:**

Systemic inflammatory response syndrome (SIRS) is a frequent complication of cardiopulmonary bypass (CPB). SIRS is associated with significant morbidity and mortality, but its pathogenesis remains incompletely understood, and as a result, biomarkers are lacking and treatment remains expectant and supportive. This study aimed to understand the pathophysiological mechanisms driving SIRS induced by CPB and identify novel therapeutic targets that might reduce systemic inflammation and improve patient outcomes.

**Methods:**

Twenty‐one patients undergoing cardiac surgery and CPB were recruited, and blood was sampled before, during and after surgery. SIRS was defined using the American College of Chest Physicians/Society of Critical Care Medicine criteria. We performed immune cell profiling and whole blood transcriptomics and measured individual mediators in plasma/serum to characterise SIRS induced by CPB.

**Results:**

Nineteen patients fulfilled criteria for SIRS, with a mean duration of 2.7 days. Neutrophil numbers rose rapidly with CPB and remained elevated for at least 48 h afterwards. Transcriptional signatures associated with neutrophil activation and degranulation were enriched during CPB. We identified a network of cytokines governing these transcriptional changes, including granulocyte colony‐stimulating factor (G‐CSF), a regulator of neutrophil production and function.

**Conclusions:**

We identified neutrophils and G‐CSF as major regulators of CPB‐induced systemic inflammation. Short‐term targeting of G‐CSF could provide a novel therapeutic strategy to limit neutrophil‐mediated inflammation and tissue damage in SIRS induced by CPB.

## Graphical abstract

Systemic inflammatory response syndrome (SIRS) is a major complication associated with cardiopulmonary bypass (CPB). In this study, we used whole blood transcriptomics to reveal neutrophils and G‐CSF as major regulators of CPB‐induced systemic inflammation. Our results suggest that short‐term targeting of G‐CSF could provide a novel therapeutic strategy to limit neutrophil‐mediated inflammation and tissue damage in SIRS induced by CPB.

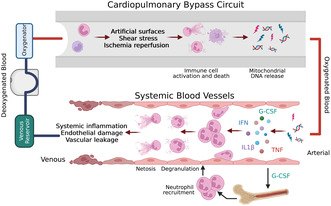

## Introduction

A sterile, systemic inflammatory response syndrome (SIRS) is a major complication of cardiopulmonary bypass (CPB). For most patients, SIRS is mild and self‐resolving; however, over 25% develop more serious features, causing significant postoperative morbidity and, in severe cases, mortality.^1^ Clinically, the most important features of SIRS are markedly reduced systemic vascular resistance and increased capillary leakage leading to fluid overload, tissue oedema and widespread organ dysfunction, especially in the lungs.^2^

SIRS is thought to be triggered by multiple mechanisms, including exposure of blood to artificial surfaces in the CPB circuit, shear stress on circulating blood cells and ischaemia–reperfusion injury to the heart following relative oxygen deprivation.^3^ SIRS is associated with activation of the coagulation, fibrinolysis, kallikrein and complement cascades.^4^ These factors are thought to combine to cause neutrophil and platelet activation and an acute, sterile inflammatory response, resulting in endothelial permeability and vascular damage.^3^

Apart from haemodynamic, respiratory and renal support, there are few effective ways to prevent or treat SIRS. Procedural modifications have been tried, including heparin‐coated bypass circuits,^5^, ^6^ intraoperative leucocyte depletion^7^ and ultrafiltration,^8^ with limited success. Pharmacologic strategies focus on coagulation, using agents, such as aprotinin, or reducing systemic inflammation with broad‐spectrum corticosteroids.^9^ These approaches are sometimes helpful but do not prevent or reverse SIRS. In fact, a recent report found that corticosteroids dampened inflammatory responses caused by CPB but had no significant impact on morbidity or mortality.^2^ There is a clear clinical imperative to better understand the mechanisms that drive SIRS induced by CPB to identify biomarkers and new therapeutic targets.

Previous studies on CPB have correlated individual proinflammatory mediators, as measured in the circulation, with clinical outcomes.^10^, ^11^ Here, we used whole blood transcriptomics and immune cell profiling to comprehensively characterise inflammatory pathways activated during and after CPB that may drive the pathophysiology of SIRS. Our analysis identifies dysregulation of the G‐CSF pathway and inappropriately activated neutrophils as promising therapeutic targets in SIRS induced by CPB.

## Results

### Patient details

Demographic and clinical characteristics of the 21 recruited patients are presented in Table 1. There were 14 men and 7 women, with an average age of 63.8 years. Most patients were undergoing coronary artery bypass graft surgery (CAGS), but other procedures included aortic valve replacement (AVR), mitral valve replacement (MVR), ascending aorta grafting and lung resection. Of the five patients requiring valve replacement surgery, none had active or treated endocarditis. Indications for aortic valve replacement included idiopathic calcification (2) and annuloaortic ectasia (1), myxomatous degeneration (1) and unspecified mitral valve disease (1). In the first 4 days after CPB, 19 patients (90%) fulfilled the criteria for SIRS and on average this lasted 2.7 days (Table 2). Assessment for SIRS was performed at the same time daily and 20 of the 21 patients were not mechanically ventilated prior to the first assessment for SIRS. Only one patient was ventilated beyond the first 24 h and remained ventilated for 8 days in total.

**Table 1:** Demographic and clinical characteristics of patients undergoing CPB

	% (*N*)
Sex	
Male	66.7% (14)
Female	33.3% (7)
Age	64 (60–72.5)
Weight (kg)	89 (75.5–106)
Height (m)	1.81 (1.67–1.74)
Medical history	
Ischaemic heart disease	81% (17)
Smoking status	Non – 38.1% (8) Ex – 52.4% (2) Current – 9.5% (11)
Hypertension	95.2% (20)
Hypercholesterolaemia	66.7% (14)
Diabetes	52.4% (11)
Previous myocardial infarction	19% (4)
Peripheral vascular disease	9.5% (2)
Preoperative atrial fibrillation	4.8% (1)
Stroke	4.8% (1)
Iron deficiency	4.8% (1)
Previous cancer	9.5% (2)
Aortic regurgitation	4.8% (1)
Dilated ascending aorta	4.8% (1)
Out‐of‐hospital ventricular fibrillation arrest	4.8% (1)
Rheumatological conditions (stable/well controlled)	19.40% (4)
Percutaneous coronary intervention	9.5% (2)
Medications	
Statins	90.5% (19)
Aspirin (anti‐platelet therapy)	90.5% (19)
Beta‐blockers	81.0% (17)
Anti‐hyperglycaemic agents	57.1% (12)
Angiotensin‐converting enzyme (ACE) inhibitors	47.6% (10)
Angiotensin receptor inhibitors	42.9% (9)
Calcium channel inhibitors	38.1% (8)
Diuretics	33.3% (7)
Proton pump inhibitors	23.8% (5)
Oral nitrates	19% (4)
Anti‐platelet agents	14.3% (3)
Anti‐depressants	14.3% (3)
Non‐steroidal analgesics	14.3% (3)
Thyroid hormone replacement	9.5% (2)
Urate lowering therapy	9.5% (2)
5 Alpha‐reductase inhibitors	4.8% (1)
Cholesterol uptake inhibitor	4.8% (1)
Preoperative blood results	
WBC (10^3^ μL^−1^)	8.28 ± 0.43
Neutrophils (10^3^ μL^−1^)	5.58 ± 0.33
Lymphocytes (10^3^ μL^−1^)	1.87 ± 0.17
Monocytes (10^3^ μL^−1^)	0.65 ± 0.05
Eosinophils (10^3^ μL^−1^)	0.42 ± 0.14
Basophils (10^3^ μL^−1^)	0.05 ± 0.01
RBCs (10^3^ μL^−1^)	4.65 ± 0.11
Platelets (10^3^ μL^−1^)	242.6 ± 12.21
HCT (%)	0.41 ± 0.01
Serum creatinine (mg dL^−1^)	85.2 ± 4.74

Values represent median (interquartile range) where data are non‐normally distributed or percentage (total number).

**Table 2:** Intraoperative and postoperative parameters

Procedure				
CAG	81.0% (17)			
AVR	14.3% (3)			
MVR	9.5% (2)			
Ascending aorta graft	4.8% (1)			
Lung wedge resection	4.8% (1)			
Repeat CAG	4.8% (1)			
Operative characteristics				
Cross‐clamp duration (min)	93 (74–109.5)			
Bypass time (min)	112 (98.5–152.5)			
	**Day 0**	**Day 1**	**Day 2**	**Day 3**
Postoperative				
Highest body temperature (°C)	37.38 ± 0.57	37.56 ± 0.39	37.48 ± 0.52	37.44 ± 0.56
Lowest body temperature (°C)	35.5 ± 2.45	36.72 ± 0.38	36.59 ± 0.48	36.6 ± 0.36
Highest respiratory rate (min)	17.5 ± 3.7	21.7 ± 3.5	21.4 ± 2.9	20.5 ± 3.7
Lowest SVRI (dynes cm^−5^)	1778 ± 617	1623 ± 332		
Highest SVRI (dynes cm^−5^)	2610 ± 627	1968 ± 454		
Maximum heart rate min^−1^	94.09 ± 16.51	100.23 ± 24.22	100.66 ± 13.73	108.8 ± 20.25
Mean heart rate min^−1^	85.14 ± 4.78	87.09 ± 5.27	86.35 ± 7.77	86.47 ± 9.61
Lowest CI (L min^−1^ m^−2^)	2.16 ± 0.43	2.74 ± 0.49	2.47 ± 0.71	
Mean CI (L min^−1^ m^−2^)	2.6 ± 0.43	3.04 ± 0.48	2.65 ± 0.77	
Peak serum creatinine (mg dL^−1^)	71.76 ± 22.96	88.8 ± 36.25	92.09 ± 37.64	93.63 ± 33.16
Leucocyte counts				
WBC (10^3^ μL^−1^)	15.87 ± 5.75	11.98 ± 3.11	12.42 ± 2.84	11.08 ± 1.8
Neutrophils (10^3^ μL^−1^)	12.55 ± 4.67	10 ± 2.92	9.5 ± 2.67	8.42 ± 1.54
Lymphocytes (10^3^ μL^−1^)	2.25 ± 2.04	1.09 ± 0.8	1.76 ± 1.49	1.88 ± 1.8
Monocytes (10^3^ μL^−1^)	1.13 ± 0.64	1.35 ± 1.56	1.6 ± 1.73	6.67 ± 22.89
Eosinophils (10^3^ μL^−1^)	0.1 ± 0.11	0 ± 0.03	0.04 ± 0.05	0.15 ± 0.22
Basophils (10^3^ μL^−1^)	0.05 ± 0.05	0.01 ± 0.04	0.01 ± 0.05	0.03 ± 0.05
RBCs (10^6^ μL^−1^)	103.42 ± 15.64	91.28 ± 11.47	82.9 ± 11.89	82.21 ± 12.1
Platelets (10^9^ μL^−1^)	170.61 ± 36.22	176.85 ± 45.58	143.95 ± 37.91	161.94 ± 48.06
HCT (%)	0.3 ± 0.04	0.27 ± 0.03	0.25 ± 0.03	0.24 ± 0.03
SIRS (no. of patients)	19 (90.4%)			
No. of patients with SIRS per day	67% (14)	67% (14)	81% (18)	57% (12)
Days with SIRS	2.7 ± 1.2			
Mechanical ventilation (h)	6.75 (IQR 6–17)			
ICU stay (h)	26 (IQR 21–45)			
Hospital stays (days)	8 (IQR 7–10)			

AVR, aortic valve replacement; CAG, coronary artery graft surgery; CI, Cardiac Index; HCT, haematocrit; MVR, mitral valve replacement; RBC, red blood cells; SIRS, systemic inflammatory response syndrome; SVRI, Systemic Vascular Resistance Index; WBC, white blood cells.

These values represent averages across the entire patient cohort. Values represent percentage (total number), mean ± standard deviation where data are normally distributed or median (interquartile range) where data are non‐normally distributed.

Three patients were returned to theatre for reasons including valve dysfunction (1), insertion of a pacemaker/implantable cardioverter defibrillator (1) or for unspecified clinical reasons (1). One patient was returned to the operating room within 24 h of the initial procedure caused by postoperative bleeding but subsequently remained haemodynamically stable on vasoconstrictor support. Clinical outcomes for our cohort of patients are detailed in Table 3 (clinical outcome definitions can be found in Supplementary table 1). No mortality was recorded within 30 days of the procedure. Three patients presented with superficial access wound infections, occurring within 30 days postoperatively. A total of 7 patients were discharged to home with no planned contact before routine review, 13 were discharged to hospital in the home with planned visits by medical staff and 1 was discharged to a rehabilitation unit.

**Table 3:** Clinical outcomes

Outcome	% (*N*)
New renal insufficiency	4.7% (1)
Peri/postoperative myocardial infarction	4.7% (1)
Peri/postoperative cardiogenic shock	4.7% (1)
New atrial fibrillation	42.7% (9)
Cardiac arrest	0% (0)
Stroke	0% (0)
Pulmonary embolism	0% (0)
Pneumonia	28.6% (6)
Wound infection	14.2% (3)
Septicaemia	4.7% (1)
Aortic dissection	0% (0)
Anticoagulant complications	0% (0)
Gastrointestinal tract complications	0% (0)
Multisystem failure	4.7% (1)
Death	0% (0)
Death within 30 days of surgery	0% (0)

### CPB causes rapid neutrophilia and increases monocytes in blood after CPB

**Figure 1:**
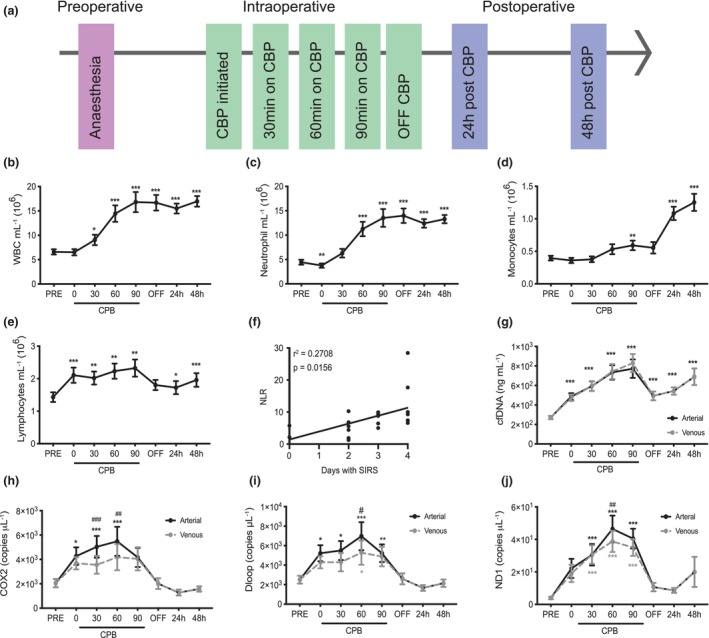
CPB induces leucocytosis and the release of extracellular DNA. **(a)** Schematic representation of the sample collection protocol. Venous blood was collected before and after CPB and blood was sampled from both the venous and arterial lines during CPB. **(b)** Quantification of white blood cells and **(c–e)** specific immune cell populations in the peripheral blood before, during and after CPB. **(f)** Correlation between NLR and number of days patients fulfilled the criteria for SIRS 24 h postop. **(g)** Quantification of cfDNA and mtDNA including **(h)** *COX2*, **(i)** *ND1* and **(j)** *D‐loop* in the plasma of patients before, during and after CPB. Data are mean ± SEM, *n* = 19. Differences are calculated compared to levels prior to surgery. *P < 0.05, **P < 0.01 and ***P < 0.001, one‐way ANOVA. ^##^ *P* < 0.01, ^###^ *P* < 0.001 for comparing venous versus arterial plasma samples.

CPB induced peripheral blood (PB) leucocytosis as early as 60 min and this typically persisted for at least 2 days after surgery (Figure 1b). This increase was mainly driven by neutrophilia (Figure 1c). In contrast, monocyte levels remained unchanged during CPB, but on average, a twofold increase in monocytes was observed in the 2 days postoperative period (Figure 1d). There was a significant increase in PB lymphocytes on CPB (Figure 1e), and while levels decreased after patients were taken off CPB, they remained higher than baseline at 24 and 48 h after surgery. At 24 h after CPB, there was a significant positive correlation between the neutrophil‐to‐lymphocyte ratio (NLR) and the number of days each patient fulfilled the criteria for SIRS (Figure 1f). This correlation was not observed at any other time point (data not shown).

### CPB induces the release of cfDNA and mtDNA

The release of cell‐associated DNA (cell‐free DNA, cfDNA) may promote inflammation.^12^ cfDNA can derive from the cell nucleus or mitochondria (mtDNA).^13^ We quantified cfDNA in plasma prior to surgery, at 30 min intervals during CPB and at defined postoperative intervals (Figure 1a). Prior to CPB, surgical trauma induced the release of cfDNA, and this was further elevated by CPB, in proportion to the length of time on CPB (Figure 1g). There was no difference in cfDNA between arterial and venous blood during surgery. Circulating cfDNA rapidly decreased after cessation of CPB although it remained higher than presurgical levels. Levels of cfDNA rose from 24 to 48 h postoperatively, although not to the extent observed with CPB. We also quantified levels of three mtDNA DNA sequences, *COX2*, *ND1* and *D‐loop*, in plasma, using droplet digital real‐time PCR (ddPCR). Data are expressed as copy number per μL; however, results were the same when analysed as fold change (data not shown). As expected, surgical trauma in the absence of CPB rapidly increased circulating *COX2*, *ND1* and *D‐loop*, but these mtDNA danger‐associated molecular patterns (DAMPs) increased further when patients were placed on CPB (Figure 1h–j). Importantly, significantly more mtDNA DAMPs were found in blood sampled from the arterial line compared with the venous line, which is the only time we observed any difference between arterial and venous blood. Given that venous blood drains from the body to the oxygenation reservoir and returns to the patient via an arterial line, these data implicate the CPB circuit as directly responsible for generating these mtDNA DAMPs. Unlike total cfDNA, levels of mtDNA DAMPs returned to baseline after cessation of CPB and remained low postoperatively (Figure 1h–j).

### Distinct transcriptomic profiles of whole blood cells collected before, during and after CPB

**Figure 2:**
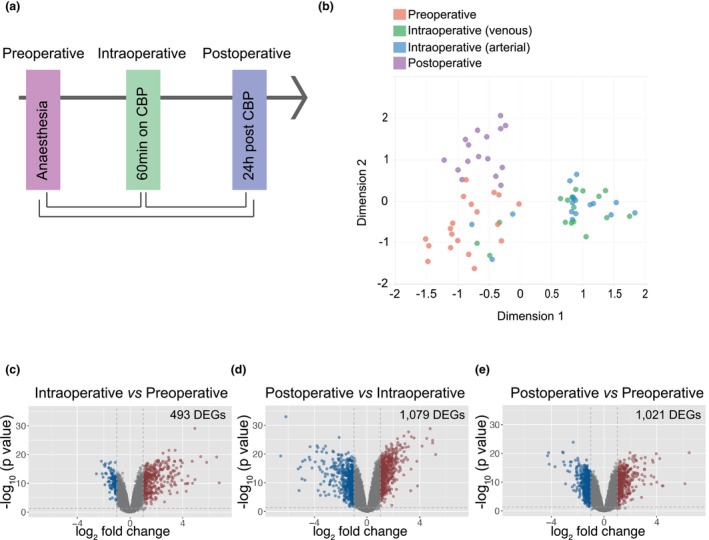
Whole blood transcriptomic profiling during and after CPB. **(a)** A schematic representation of sample collection. **(b)** Multidimensional scaling (MDS) plot of whole blood transcriptomic profiles before surgery (preoperative), from the venous and the arterial lines 60 min into CPB (intraoperative) and 24 h after CPB (postoperative) (*n* = 21). **(c–e)** Quantitative comparison of differentially expressed genes before, during and after CPB, where the volcano plot represents relative gene expression changes. Red dots indicate transcripts upregulated with a multicomparison adjusted −log_10_ (*P*‐value) ≥ 1.3 and a linear fold change ≥ 2; blue dots indicate transcripts downregulated with a multicomparison adjusted −log_10_ (*P*‐value) ≥ 2 and a linear fold change ≤ −2; grey dots indicate genes that do not meet either criterion. Samples from individuals who developed SIRS for ≥ 1 days (*n* = 19) were included in the differential gene expression analysis.

Transcriptomic analysis was performed on whole blood cells collected prior to surgery (preoperative), 60 min after the initiation of CPB (intraoperative) and 24 h after surgery (postoperative, Figure 2a). Venous blood was sampled pre‐ and postoperatively and both arterial and venous blood samples were collected intraoperatively. After filtering for low‐expression and non‐coding genes, a total of 12 018 genes were retained for differential expression analysis. A total of 1259 genes were identified as differentially expressed between the groups examined, using a false discovery rate (FDR) cut‐off of 0.05 and fold change (FC) cut‐off threshold of 2 (Supplementary table 2). Unsupervised clustering analysis using multidimensional scaling revealed a significant change in the preoperative transcriptomic profile in whole blood during and after CPB (Figure 2b).

Clustering analysis was performed based on the transcriptomic profile of these samples. This analysis revealed significant changes to the preoperative transcriptomic profile in whole blood during and after CPB (Figure 2b). However, there were no significant differences in the transcriptomic profile of whole blood collected from venous and arterial lines during CPB and as such these samples were pooled for analysis.

We compared pre‐ versus intra‐ versus postoperative samples to provide an overview of the differentially regulated genes and specific pathways that become activated in response to CPB. First, we compared pre‐ and intraoperative blood and found 493 genes were differentially expressed (with an absolute fold change > 2, FDR < 0.05), 369 genes were upregulated and 124 were downregulated in the intraoperative samples (Figure 2c). Canonical pathway analysis predicted that the upregulated genes were associated with airway pathology in chronic obstructive pulmonary disease, granulocyte adhesion and diapedesis and IL‐17 and IL‐6 signalling (Table 4). Downregulated genes were predicted to be involved in phagosome formation, neuroinflammation and G‐protein‐coupled receptor signalling. A total of 1079 genes were found to be differentially regulated between the intraoperative and postoperative samples (Figure 2d). A total of 529 genes upregulated after surgery and pathway analysis suggested these genes were involved in phagosome formation, the inflammasome pathway and pyroptosis (Table 4). A total of 550 genes were downregulated and predicted to be involved in Th1 and Th2 activation pathways, cytokine production and granulocyte adhesion and diapedesis.

**Table 4:** Predicted biological pathways upregulated in response to CPB

Upregulated DEG		Downregulated DEG	
Ingenuity canonical pathways	−log (*P‐*value)	Ingenuity canonical pathways	−log (*P*‐value)
**Preoperative versus Intraoperative**			
Airway pathology in chronic obstructive pulmonary disease	7.62	Phagosome formation	4.86
Granulocyte adhesion and diapedesis	6.00	Neuroinflammation signalling pathway	4.39
Role of IL‐17A in psoriasis	5.90	Gαs signalling	4.32
Tumor microenvironment pathway	5.52	Colorectal cancer metastasis signalling	4.07
Osteoarthritis pathway	4.88	G‐protein‐coupled receptor signalling	3.54
Hepatic fibrosis signalling pathway	4.69	Sperm motility	3.43
Role of IL‐17A in arthritis	4.67	Endothelin‐1 signalling	3.30
IL‐6 signalling	4.65	Macropinocytosis signalling	3.18
Wound healing signalling pathway	4.56	TREM1 signalling	3.16
PPAR signalling	4.49	Gαi signalling	3.09
**Intraoperative versus Postoperative**			
Phagosome formation	11.60	Airway pathology in chronic obstructive pulmonary disease	7.21
Osteoarthritis pathway	7.83	Th1 and Th2 activation pathway	6.47
Colorectal cancer metastasis signalling	7.39	Sperm motility	5.29
Gαs signalling	6.95	Crosstalk between dendritic cells and natural killer cells	5.23
G‐protein‐coupled receptor signalling	6.84	Differential regulation of cytokine production in intestinal epithelial cells by IL‐17A and IL‐17F	5.06
Inflammasome pathway	6.84	Th2 pathway	4.90
CREB signalling in neurons	6.14	Role of hypercytokinaemia/hyperchemokinaemia in the pathogenesis of influenza	4.66
Breast cancer regulation by stathmin1	5.44	Granulocyte adhesion and diapedesis	4.62
Pyroptosis signalling pathway	5.20	Atherosclerosis signalling	4.41
Macropinocytosis signalling	5.19	Pulmonary fibrosis idiopathic signalling pathway	4.30
**Preoperative versus Postoperative**			
Phagosome formation	10.70	Th1 and Th2 activation pathway	11.60
Osteoarthritis pathway	7.97	Th2 pathway	11.00
Eicosanoid signalling	6.46	Th1 pathway	8.40
IL‐10 signalling	6.23	CREB signalling in neurons	6.88
Atherosclerosis signalling	6.17	Sperm motility	6.76
Wound healing signalling pathway	6.13	Natural killer cell signalling	6.60
HIF1α signalling	5.94	IL‐23 signalling pathway	4.99
Role of macrophages, fibroblasts and endothelial cells in rheumatoid arthritis	5.67	nNOS signalling in skeletal muscle cells	4.85
LXR/RXR activation	5.66	Hepatic fibrosis signalling pathway	4.64
Hepatic fibrosis/hepatic stellate cell activation	5.63	IL‐15 production	4.48

CREB, cAMP response element‐binding protein; G protein, guanine nucleotide‐binding proteins; Gαi, Gᵢ protein alpha subunit; Gαs, Gs alpha subunit; HIF1α, hypoxia‐inducible factor 1‐alpha; IL, interleukin; LXR, liver X receptor; nNOS, neuronal nitric oxide synthase; PPAR, peroxisome proliferator‐activated receptors; RXR, retinoid X receptor; Th, T helper; TREM1, triggering receptor expressed on myeloid cells 1.

Comparison of transcriptional changes between pre‐ and postoperative samples identified 436 upregulated and 585 downregulated genes in the postoperative samples (Figure 2e). Pathways upregulated after surgery included those related to phagosome formation, IL‐10 signalling and wound‐healing signalling pathways. Downregulated genes were associated with Th1 and Th2 activation pathways, IL‐23 and natural killer cell signalling (Table 4).

### CPB activates the molecular profiles of granulocyte adhesion and diapedesis

**Figure 3:**
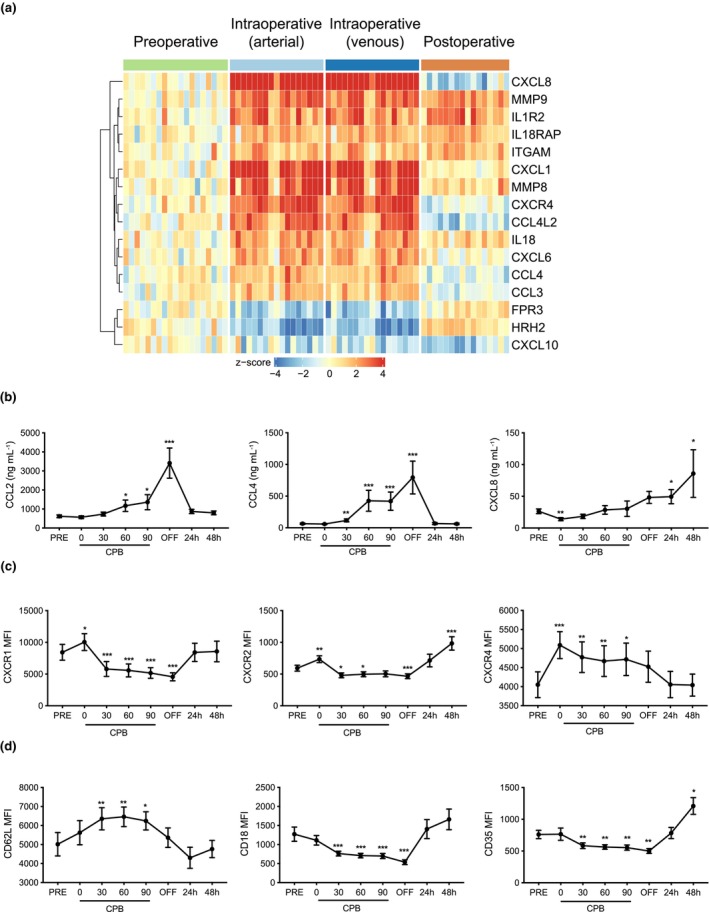
CPB induces mediators associated with granulocyte activation, diapedesis and migration. **(a)** A heatmap showing the expression profile of transcripts involved in granulocyte adhesion and diapedesis that were significantly upregulated (red) or downregulated (blue) in whole blood cells during CPB compared to preoperative levels. **(b)** Serum concentrations of CCL2, CCL4 and CXCL8 were measured before, during and after CPB according to the schematic in Figure 1a. **(c)** Membrane expression of chemokine receptors CXCR1, CXCR2 and CXCR4 on neutrophils. **(d)** Expression of neutrophil activation markers CD62L, CD16 and CD35 assessed by flow cytometry on samples taken before, during and after CPB, represented as MFI. Data are mean ± SEM. *P < 0.05, **P < 0.01 and ***P < 0.001, one‐way ANOVA, *n* = 19.

We sought to identify mechanisms contributing to systemic inflammation induced by CPB and therefore focussed on pathways activated intraoperatively. Whole blood cell transcriptomics revealed changes in genes associated with granulocyte adhesion and diapedesis. CPB induced increased expression of neutrophil chemoattractants, including CXCL1, CXCL4, CXCL8 and CCL4^14^ (Figure 3a). There was increased expression of matrix metalloproteinases 8 and 9, which are known to facilitate neutrophil migration through the extracellular matrix^15^, ^16^ (Figure 3a). These transcriptional changes were consistent with the neutrophilia we observed in the PB during and after CPB (Figure 1c). The highest expression of these gene transcripts was observed intraoperatively, with levels decreasing 24 h after surgery, suggesting that CPB itself is responsible for the transcriptional changes. We also measured serum concentrations of chemokines known to induce neutrophil recruitment, including CCL2, CCL4 and CXCL8 (IL‐8). In general, serum protein levels of these mediators lagged behind transcriptional changes, consistent with CPB‐induced production. Serum CCL2 and CCL4 rose steadily in response to CPB, with levels increasing over time (Figure 3b). Both CCL2 and CCL4 peaked immediately after patients were removed from CPB and both chemokines returned to baseline within 24 h, remaining low at 48 h after surgery. In contrast, there was a rise in serum concentrations of CXCL8 during and just after CPB, but the highest levels were found 24 and 48 h after surgery (Figure 3b).

Flow cytometry was used to assess expression of chemokine receptor family members on neutrophils before, during and after CPB. CXCR1 and 2 displayed a small but significant increase in response to surgical trauma, independent of CPB (Figure 3c). After patients were connected to CPB, expression of these chemokine receptors decreased below baseline. CXCR1 returned to baseline levels 24 h after CPB, while CXCR2 remained significantly increased above baseline 48 h after surgery. In contrast, CXCR4 expression on neutrophils increased over baseline with commencement of surgery, and its expression remained elevated throughout CPB (Figure 3c). Similar to CXCR1, CXCR4 expression returned to baseline in the postoperative period.

We also assessed the expression of other neutrophil activation and adhesion markers. CD62L expression was significantly higher on neutrophils during CPB and returned to baseline levels after CPB (Figure 3d). In line with previous studies showing CD18 and CD35 decrease with inflammation, we observed rapid reduction in expression of these neutrophil markers with CPB. CD18 levels returned to normal during the postoperative period, but CD35 expression was significantly increased at 48 h after surgery (Figure 3d). Taken together, these results suggest that CPB induces production of neutrophil‐associated chemokines as well as affecting expression of surface molecules involved in neutrophil activation and trafficking during inflammation.

### CPB induces neutrophil degranulation and release of proteases into the circulation

**Figure 4:**
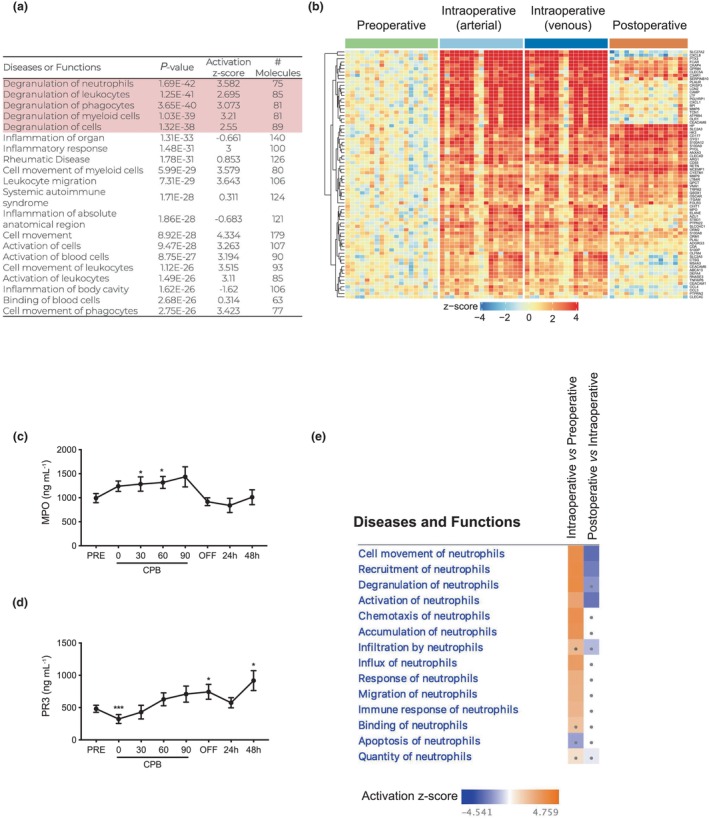
CPB triggers activation and degranulation of neutrophils. **(a)** Table 1 represents the top 20 canonical disease and functional pathways in whole blood cells before and during CPB. **(b)** A heatmap of differentially expressed transcripts involved in degranulation of neutrophils during CBP. Quantification of serum levels of **(c)** MPO and **(d)** PR3 before, during and after CPB. **(e)** Comparison of the top biological pathways related to neutrophil function that are predicted to be altered based on the genes differentially expressed intraoperatively compared to preoperatively (first column), and postoperatively compared to intraoperatively (second column). The predicted direction of change (activation or repression) is represented as the activation *z*‐score. The dots indicate pathways with an absolute activation *z*‐score < 2. Data are mean ± SEM, *P < 0.05, **P < 0.01 and ***P < 0.001, one‐way ANOVA, *n* = 19.

In addition to transcriptional changes consistent with increased neutrophil recruitment from the BM into PB, transcriptional signatures associated with degranulation were also increased by CPB (Figure 4a). A total of 75 genes linked to neutrophil degranulation were upregulated during CPB compared to preoperatively (Figure 4b). Expression of the majority of these genes returned to baseline 24 h after CPB, but a small subset remained elevated 24 h after CPB. Pathways associated with neutrophil activation and degranulation were highly represented in intraoperative blood samples and downregulated by 24 h postoperatively (Figure 4e). In keeping with these transcriptional signatures, there was a significant increase in serum levels of neutrophil granule proteins myeloperoxidase (MPO) and proteinase 3 (PR3) when patients were on CPB. Serum MPO returned to baseline 30 min after normal blood circulation was restored, whereas PR3 was significantly increased 48 h after CBP (Figure 4c–e).

### A network of cytokines controls the transcriptional changes observed in response to CPB

**Figure 5:**
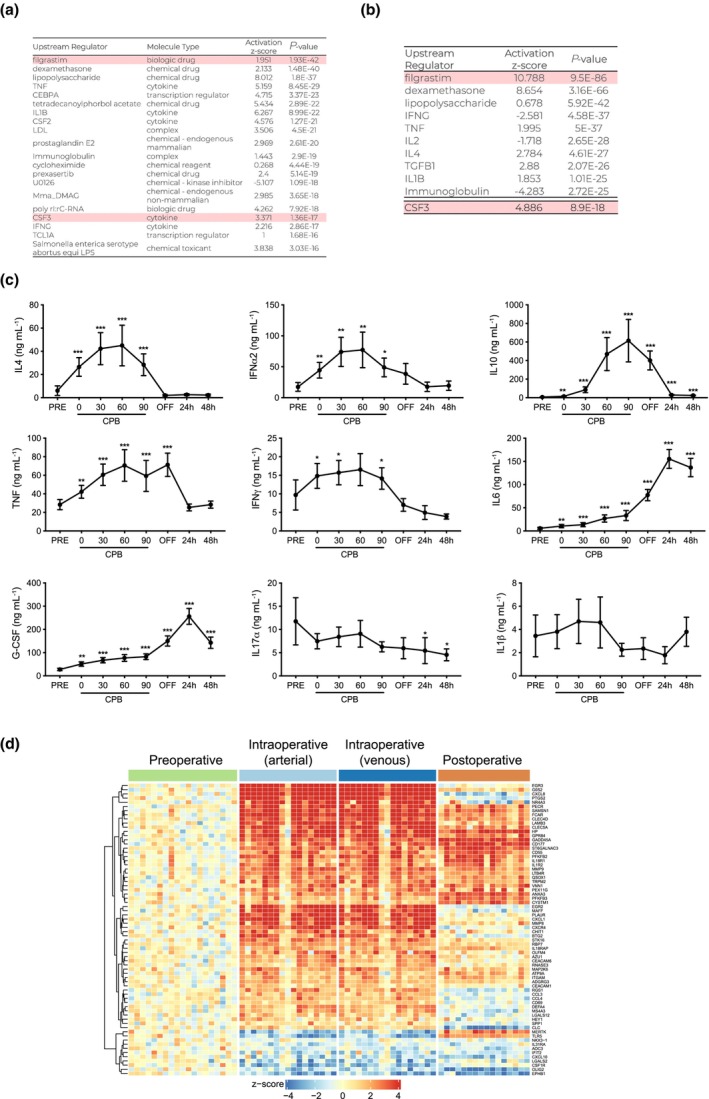
G‐CSF is a key driver of the transcriptional changes that occur during and after CPB. Top 20 upstream regulators identified based on gene transcript expression analysis of whole blood cells during **(a)** and after CPB **(b)** compared with preoperative samples. **(c)** Quantification of cytokines produced in response to CBP (*n* = 19). **(d)** A heatmap of differentially regulated gene transcripts associated with G‐CSF, where upregulated genes are shown in red and downregulated genes in blue. Data are mean ± SEM, *P < 0.05, **P < 0.01 and ***P < 0.001, one‐way ANOVA.

Pathway analysis was performed to search for upstream regulators responsible for the gene expression changes induced by CPB in peripheral blood cells. Granulocyte colony‐stimulating factor (G‐CSF), TNF, IL1β and IFNγ were identified as drivers of differential gene expression both during CPB (Figure 5a) and 24 h postsurgery (Figure 5b) compared to the preoperative state. IL2, IL4 and TGFβ1 were predicted to mediate transcriptional changes postoperatively (Figure 5b). We therefore quantified serum cytokines and observed distinct temporal profiles induced by CPB. A wave of cytokines was rapidly induced immediately following the commencement of surgery, including IL4, IFNα2, IL10, TNF and IFNγ, and these cytokines further increased when patients were placed on CPB (Figure 5c). Levels of these cytokines peaked on CPB, and returned to baseline within 24 h, suggesting CPB drives production. Notably, while serum IL10 decreased in the days following surgery, it remained above baseline. In contrast, G‐CSF and IL6 rose steadily in response to CPB but did not peak until 24 h postoperatively (Figure 5c). Both cytokines then decreased from 24 to 48 h after surgery but remained significantly higher than baseline levels at 48 h. Similar to chemokines, serum cytokine levels lagged behind the transcriptional changes. IL17α and IL1β were detectable, but no significant serial changes were found during or after CPB (Figure 5c). There was no apparent correlation between the levels of these individual cytokines and chemokines and clinical parameters, the severity of SIRS or clinical outcomes.

### G‐CSF is a key upstream regulator of the transcriptional changes induced by CPB

Our analysis revealed that G‐CSF is predicted to be a key driver of the gene expression changes that occur in PB cells during and after CPB (Figure 5a and b). A total of 72 G‐CSF‐regulated genes were increased during CPB and expression of 30 of these genes remained high for at least 24 h postoperative (Figure 5d). Consistent with this prediction, serum G‐CSF increased at the commencement of surgery, and levels rose steadily on CPB (Figure 5c). Serum G‐CSF peaked 24 h after CPB and decreased by 48 h, although on average it remained up to 5× higher than baseline.

## Discussion

Systemic inflammation induced by CPB represents a major clinical challenge. While supportive management has improved, to date no interventions have had a significant impact on preventing or reversing the progression of SIRS. In this study, we used whole blood transcriptomics to identify neutrophils and G‐CSF as key mediators of systemic inflammation induced by CPB. Given its role as a key regulator of neutrophil production, release and function, we propose that careful targeting of the G‐CSF pathway could prevent excessive neutrophil activation during CPB and reduce postoperative morbidity and mortality from SIRS.

It is well established that CPB causes neutrophilia, indeed it has been hypothesised that the inflammation induced by CPB may be mediated by neutrophil activation.^17^, ^18^, ^19^, ^20^ However, what drives the recruitment of neutrophils into the circulation and what governs neutrophil activation during CPB remain poorly understood. To address this, we performed global transcriptomics in peripheral blood cells, which revealed increased expression of genes associated with neutrophil adhesion and diapedesis, including chemokines, adhesion molecules and enzymes that allow neutrophil migration through the extracellular matrix.^15^, ^16^ We then assessed serum levels of neutrophil chemoattractants CCL2, CCL4 and CXCR8 and found these were indeed increased during and after CPB. We also observed modulation of chemokine receptors CXCR1, 2 and 4 on the surface of neutrophils after patients were placed on CPB. These receptors control the egress of neutrophils from the bone marrow into the circulation, as well as their recruitment to sites of inflammation^14^ and changes in their expression likely contribute to the profound neutrophilia induced in response to CPB. CPB caused significant changes in neutrophil activation markers. Levels of CD62L increased on CPB and decreased immediately after normal blood flow was restored, while CD18 and CD35 decreased in response to CBP and returned to baseline 24 h after surgery. CD62L is a cell surface adhesion molecule involved in neutrophil chemotaxis and adhesion to the endothelium.^21^ CD18 can be expressed at the membrane and released in a soluble form in response to inflammatory signals, including TNF.^22^ CD35 is a complement receptor reported to be decreased during inflammation.^23^

In keeping with increased neutrophil recruitment and activation, transcriptional signatures associated with neutrophil degranulation were also enriched during CPB. Neutrophil granules contain enzymes and proteases with potent cytotoxic activity that are released into neutrophil phagosomes and also the extracellular environment.^24^ While essential for host defence, release of these granule proteins in the context of sterile inflammation (as in CPB) can cause endothelial and tissue damage.^24^ In keeping with increased expression of genes associated with neutrophil degranulation, serum concentrations of neutrophil granule proteins MPO and PR3 rose during CPB. The transcriptional signatures associated with neutrophil degranulation and serum levels of granule proteins returned to baseline when normal blood circulation was restored. These findings strongly suggest that neutrophil degranulation is caused by the CPB circuit itself. However, we cannot rule out the possibility that surgical trauma also induces the release of toxic neutrophil granule proteins into the circulation, and future studies comparing coronary artery bypass graft surgery (CABG) performed on‐ and off‐pump would more directly address this.

Our results are in keeping with studies showing that neutrophils are key mediators of inflammation and endothelial cell damage induced by CPB.^17^, ^18^, ^19^, ^20^ Importantly, our whole blood transcriptomics analysis identified G‐CSF as the most significant upstream regulator of the transcriptional changes observed during and after CPB. Serum G‐CSF increased early in CPB, and this was tracked with neutrophilia. G‐CSF is essential for the production, differentiation and release of mature neutrophils from the bone marrow, and it contributes to their activation. While barely detectable in the serum of healthy individuals, serum G‐CSF increases in infection and inflammatory diseases, when it facilitates rapid mobilisation of neutrophils into the circulation.^25^ G‐CSF can also modulate the function of mature neutrophils, promoting transendothelial migration and enhancing production of reactive oxygen species and the generation of NETs (neutrophil extracellular traps).^25^

To date, there is no effective treatment for SIRS, apart from supportive therapy, and the mortality rate remains about 5%.^2^ Our study provides strong rationale for limiting neutrophilia and neutrophil hyperactivation induced by CPB in order to reduce the incidence and severity of SIRS. We previously reported that G‐CSF receptor (G‐CSFR) antagonism can prevent inflammation‐induced neutrophilia and limit the progression of an experimental model of rheumatoid arthritis.^26^, ^27^ More recently, an anti‐G‐CSFR antibody has undergone clinical trials for treatment of neutrophilic dermatoses (clinicaltrial.gov identified: NCT03972280, NCT04570267). To date, results show that blockade of endogenous G‐CSFR is well tolerated, potently inhibits G‐CSF‐induced neutrophilia and counteracts the associated whole blood transcriptomic changes.^27^, ^28^ Importantly, treatment with the G‐CSFR inhibitory antibody did not induce clinically significant neutropenia, or inhibit neutrophil effector functions.^27^ Given the vast majority of CPB procedures are sterile, and patients are closely monitored during the peri‐operative period for infection, we suggest G‐CSFR inhibition could provide a mechanism‐based approach to limit neutrophil‐mediated inflammation and organ damage.

In addition, granulopoiesis is not solely driven by G‐CSF, with contributions from other cytokines, such as IL‐6 and GM‐CSF.^29^, ^30^, ^31^, ^32^ Blockade of other regulators of neutrophil production, release or activation may also be effective in limiting CPB‐induced SIRS. For example, CXCR1 and 2 are chemokine receptors that control the egress of neutrophils from the bone marrow and migration into sites of inflammation.^33^, ^34^ Inhibitors to CXCR1 and 2 are effective in controlling neutrophil‐mediated inflammation in various animal models of disease.^35^ Several CXCR1/2 inhibitors are now undergoing clinical trials for cystic fibrosis, chronic obstructive pulmonary disease (COPD) and ulcerative colitis.^35^

Our study reveals a network of cytokines in addition to G‐CSF that drive transcriptional changes in whole blood cells in response to CPB. Previous studies demonstrate that CPB elevates serum cytokines,^10^, ^11^, ^36^, ^37^, ^38^ but our study provides a comprehensive transcriptional and proteomic analysis in patients on CPB and the days following surgery. We found that CPB induces cytokines in distinct temporal patterns. We infer the first wave is directly induced by CPB because levels peaked during surgery and rapidly returned to baseline after blood flow was restored. This first wave includes proinflammatory cytokines G‐CSF, IFNα2 and TNF, as well as anti‐inflammatory cytokines IL10 and IL4. IL4 is a pleotropic cytokine which exerts effects on both haematopoietic and non‐haematopoietic cells. It can stimulate B cell and T cell proliferation, promote differentiation of naive T cells into T helper 2 effector cells and inhibit the secretion of the proinflammatory cytokines, including IL1, IL6 and TNF.^39^ Expression of IL4 is increased in animal models of cardiac injury^40^, ^41^ and in cardiac fibrosis^42^ and IL4 can enhance collagen production by cardiac fibroblasts through the STAT6 signalling pathway.^43^ A second wave of cytokines peaks postoperatively, including IL6 and G‐CSF. Our study strongly suggests a network of transcriptionally regulated cytokines controls the inflammatory response induced by CPB, providing a range of potential therapeutic targets, in addition to G‐CSF.

Emerging data suggest that the release of DNA extracellularly can promote inflammation^12^, ^44^ and cfDNA may represent a biomarker for inflammation and tissue damage. Surgical trauma and the associated tissue injury also induce a systemic inflammatory response. Levels of cfDNA predicted mortality rates in infants undergoing cardiac surgery.^45^, ^46^, ^47^ cfDNA can derive from the cell nucleus or mitochondria.^13^ While both sources can be proinflammatory, mtDNA are well‐characterised DAMPs that can mediate innate immune responses.^48^ Previous studies have focussed on the levels of cfDNA after surgery, but we demonstrate that circulating cfDNA levels peak during CPB and increase the longer patients remain on CPB. Restoration of normal blood flow resulted in a prompt decrease in plasma cfDNA, suggesting CPB itself drives the release of extracellular DNA. This was further supported by the observation that mtDNA DAMPs were higher in arterial blood exiting the CPB circuit, compared to venous blood collected from the body. Why CPB induces the release of extracellular DNA, which cell types release it and how this contributes to SIRS is currently unknown. We suggest the release of extracellular DNA may relate to neutrophil cell activation, damage or death induced by exposure to foreign surfaces in the CPB circuit or artificial sheer stress,^49^, ^50^ endothelial cell damage^4^, ^51^ or surgical trauma.^52^ It is possible that cf and mtDNA promote proinflammatory cytokine production by activating the NOD‐, LRR‐ and pyrin domain‐containing protein 3 (NLRP3) inflammasome or toll‐like receptor 9 (TLR) signalling pathway^44^ or trigger the release of NETs.^46^ Consistent with increased cellular activation by DAMPs, we observed an intraoperative rise in the levels of the type 1 interferon, IFNα2, which peaked rapidly, and then returned to baseline levels after normal blood flow was restored.^53^ Intriguingly, administration of a DNA‐degrading enzyme (DNaseI) in a rat model of CPB effectively reduced plasma cfDNA levels, decreasing endothelial dysfunction and reducing systemic inflammation.^54^ Taken together, we suggest that DNase1 could represent another potential therapeutic approach to limiting SIRS.

Currently, there are few useful biomarkers that distinguish patients with mild and self‐resolving SIRS from those more likely to develop severe SIRS.^11^ In our patient cohort, we observed a positive correlation between the NLR 24 h after surgery and the number of days patients fulfilled criteria for SIRS. This is consistent with previous studies showing an elevated NLR in the postoperative period is associated with increased length of ICU and hospital stays, plus higher mortality rates after CPB.^55^ NLR can predict outcomes in other cardiovascular conditions including acute heart failure^56^ and infective endocarditis^57^ and could provide a routinely available and inexpensive tool to identify patients at risk of developing severe SIRS after CPB. We also found that CPB causes the inappropriate activation of neutrophils, possibly driven by dysregulated production of G‐CSF. If these findings are confirmed, assessing serum cytokines and neutrophil activation markers, as well as the NLR, may be introduced into future clinical practice.

While not sufficiently powered to identify novel biomarkers that predict the severity of SIRS, our comprehensive profiling demonstrated that global transcriptomics on whole blood cells can be used to identify differentially expressed genes, molecular pathways and cytokine associated with CPB. This study was not the first to use whole blood transcriptomics to identify genes involved in CPB.^58^ However, our study included a larger patient cohort and more extensive sampling, where blood was collected before, during and after surgery. This allowed us to comprehensively characterise inflammatory pathways activated by CPB and compare these in the same patient 24 h later. Using this strategy, we identify G‐CSF as a major regulator of transcriptional changes in the peripheral blood during CPB. Complementing the whole blood cell transcriptomics, our study included a comprehensive analysis of immune cells and serum factors before, at various time points during, and in the 2 days after CPB, providing a more complete characterisation of the changes induced by CPB. Using this approach, we reveal a network of cytokines, including G‐CSF, and the release of cell‐free DNA that are likely to drive these transcriptional changes, offering new therapeutic targets for CPB‐induced SIRS.

This was a pilot, hypothesis‐generating study and, as such, had a relatively small sample size. Only two patients in the cohort did not meet criteria for SIRS within 4 days postsurgery and the study was not powered to show a correlation between activation of the G‐CSF pathway and the occurrence or severity of SIRS. Another limitation of this study is the absence of an ‘off‐pump’ control surgical group. CABG usually involves CPB; however, it can be performed ‘off‐pump’, with the heart continuously beating and providing normal blood flow throughout the procedure.^59^ Patients undergoing off‐pump CAGB may have less systemic inflammation, including significantly reduced levels of TNF, IL‐8 and IL‐6 and complement factor C3a, compared to CPB.^60^, ^61^ However, off‐pump CABG does not completely prevent a systemic inflammatory response. While not the focus of the present study, comparing off‐ versus on‐pump CABG in terms of the G‐CSF pathway would help dissociate the contribution of surgical trauma alone from CPB.

## Conclusions

We have identified neutrophils and G‐CSF as major regulators of CPB‐induced systemic inflammation. Measuring the NLR, neutrophil activation and DAMPs could provide new biomarkers for SIRS. Careful, short‐term targeting of G‐CSF could provide a novel therapeutic strategy to limit neutrophil‐mediated inflammation and tissue damage in SIRS induced by CPB.

## Methods

### Patient recruitment

This prospective, observational pilot study was approved by the Human Research and Ethics Committees of Melbourne Health (HREC/16/MH/16) and the Walter and Eliza Hall Institute of Medical Research (G16/08). Written informed consent was obtained from all participants and the study was conducted in accordance with ethical standards laid down in the Declaration of Helsinki of 1975, as revised in 1983. All patients aged over 18 years scheduled to undergo elective CPB surgery at the Royal Melbourne Hospital were eligible for enrolment. Exclusion criteria included active endocarditis, off‐pump surgery, pregnancy or an inability to provide informed consent. While moderate‐to‐deep hypothermia was not a specific exclusion criterion, patients in our cohort were not subjected to hypothermic conditions during the surgery. Patients were recruited consecutively, and 21 consented during the timeframe. The demographic and clinical characteristics of patients enrolled in this study are presented in Table 1.

### Human sample collection

Preoperative blood samples were obtained in the 3 days before surgery and postop blood samples were collected daily (before 12 pm). To perform immune cell profiling and assess plasma proteins, EDTA‐anticoagulated blood was collected pre‐, intra‐ and postoperatively (at 24 and 48 h) from individual patients (Figure 1a). Both venous and arterial blood were collected at 30 min intervals while patients were on CPB and 30 min after the restoration of normal blood flow (Figure 1a). Venous blood was collected pre‐ and postoperatively. Plasma was separated by centrifugation at 1500 *g* for 15 min, followed by centrifugation at 3500 *g* in a fresh tube for 15 min to remove platelets. Plasma samples were stored at −80°C until analysis. For serum, blood was allowed to coagulate for 1 h before centrifugation at 1500 *g* for 15 min. Serum samples were stored at −80°C. For whole‐blood transcriptomic analysis, venous blood was sampled pre‐ and postoperatively and venous and arterial blood was collected after 60 min on CPB (Figure 2a). Blood was collected into PAXgene Blood RNA collection tubes (PreAnalytiX GmbH, Hombrechtikon, Switzerland), which were stored at −20°C until RNA was extracted.

### Data collection

Clinical data relating to preoperative, intraoperative and postoperative variables were obtained from patients enrolled in the study. Preoperative data included patient demographics, surgical indication and procedure, medical history and preoperative medications, presented in Table 1. No patients were receiving corticosteroids or other immunosuppressive medications at the time of surgery or in the postoperative period, and most were on standard management of co‐morbidities including hypertension, hyperlipidaemia, atherosclerosis and type 2 diabetes. Intraoperative data collected included time on CPB, cross‐clamp time and use of blood products. Postoperative data included laboratory tests, ventilation requirements and clinical outcomes. Intraoperative and postoperative variables are presented in Table 2. No specific temperature control strategy was applied within the indexed postoperative period and patients did not receive corticosteroids or beta‐blockade during this time. Four patients received low‐dose dobutamine postoperatively and five patients required low‐dose noradrenaline to maintain a mean blood pressure of over 70 mmHg.

### Classification of SIRS

The assessment for SIRS was performed at the same time daily for all patients (between 11 am and 12 pm). SIRS was diagnosed if two or more of the following criteria were met: tachycardia (heart rate > 90 beats min^−1^), tachypnoea (respiratory rate > 20 breaths min^−1^), fever or hypothermia (temperature > 38 or < 36 °C) and increased white cell count (over 12 000 cells mm^−3^, or more than 10% immature neutrophils).^62^ SIRS was assessed for the first 4 days postsurgery. Only patients who met the criteria for SIRS for 1 or more days were included in the analysis. Based on this, a total of 19 patients were included and 2 patients who did not develop SIRS were excluded.

### Immune cell profiling of whole blood

Whole blood was stained with the following antibodies: CD11b (ICRF44), CD14 (M5E2), CD16 (3G8), G‐CSFR (CSL324), CD62L (DREG‐56), CD66b (G10F5), CD18 (6.7), CD35 (E11), CXCR1 (8F1/CXCR1), CXCR2 (5E8/ CXCR2), CXCR4 (12G5), CD3 (SK7) and CD19 (HIB19) (BD Biosciences, Franklin Lakes, USA). Flow cytometry data were acquired with a MACSQuant Analyser (Miltenyi Biotec, Bergisch Gladbach, Germany) and analysed with Flowjo software (BD Biosciences, Franklin Lakes, USA).

### Serum cytokine levels

The concentrations of cytokines and chemokines in PB were measured using MILLIPLEX MAP assays (Merck Millipore, Burlington, USA), according to the manufacturer's instructions.

### Quantification of plasma cell‐free (cf) DNA

Plasma levels of cfDNA were quantified with the Quant‐iT PicoGreen dsDNA assay (Life Technologies, Carlsbad, USA) according to the manufacturer's instructions.

### Detection of plasma mitochondrial (mt) DNA

Droplet digital polymerase chain reaction (ddPCR) was used to detect cyclooxygenase 2 (COX2), NADH–ubiquinone oxidoreductase chain 1 (ND1) and displacement loop (D‐loop) in DNA extracted from plasma samples using the BioRad QX200 Automated Droplet Generator, BioRad C1000 Touch Thermal Cycler and QX200 Automated Droplet Reader. Primer sequences for ddPCR analyses of the indicated sequences can be found in Supplementary table 3.

Droplet digital polymerase chain reaction (ddPCR) was used to detect cyclooxygenase 2 (COX2), NADH–ubiquinone oxidoreductase chain 1 (ND1) and displacement‐loop (D‐loop) in DNA extracted from plasma samples using the BioRad QX200 Automated Droplet Generator, BioRad C1000 Touch Thermal Cycler and QX200 Automated Droplet Reader. Primer sequences for qRT‐PCR analyses of the indicated sequences can be found in Supplementary table 3.

### Transcriptome analysis

Whole blood total RNA was extracted using the PAXgene blood RNA extraction kit (Qiagen, Hilden, Germany), according to the manufacturer's instructions. Extracted RNA samples were quality checked using the Agilent 2100 Bioanalyzer platform (Agilent Technologies, Santa Clara, USA). cDNA libraries were prepared for sequencing using strand‐specific TrueSeq kits, with ribosomal RNA and globin mRNA depletion. At least 30 million 150 bp paired‐end reads were generated per sample using Illumina NovaSeq 6000 (Illumina, San Diego, USA), employing standard protocols at the Victoria Clinical Genetics Services facility (Melbourne, Australia). Images were analysed in real time by the NovaSeq Control Software v1.6.0 and Real Time Analysis 3 (RTA3) software v3.4.4. RTA performs real‐time base calling on the NovaSeq instrument. The Illumina bcl2fastq 2.20.0.422 pipeline was then used to generate sequence data. Data are available from the authors upon request.

### Bioinformatics analysis

Sequenced reads were mapped to human reference genome GRCh38.p12 using Star (v2.6.1)^63^ with default parameters, except for the quantMode TranscriptomeSAM setting. Reads were summarised at the gene level using the featureCounts tool from the Subread package (v1.6.4),^64^ in a strand‐specific manner. Differential expression was analysed using limma (v3.40.6) from the bioconductor package, following a published workflow.^65^ Lists of differentially expressed (DE) genes were used to perform pathway analysis with the Ingenuity Pathway Analysis (IPA) software (Qiagen, Hilden, Germany). The core analysis was performed on each gene list separately using the default settings. Cut‐off values of fold change and *P*‐values were applied as specified in the text. The top‐ranked canonical pathways, diseases and functions and upstream regulators were selected to derive biological interpretations of the transcriptome analysis.

### Statistical analyses

Statistical differences between time points were analysed using the linear mixed effect (LME) model. The log_10_ transformation was applied to the data to fulfil underlying assumptions of the LME model. Dunnett's multiple‐comparison test was used to compare each time point to the baseline. Spearman's correlation was used to measure the correlation between neutrophil–lymphocyte ratio (NLR) and the number of days a patient fulfilled the criteria for SIRS. All analyses were performed using GraphPad Prism version 9 (GraphPad Software). Data are shown as means ± SEM unless stated otherwise. Levels of statistical significance are expressed as *P‐*values: *P < 0.05, **P < 0.01 and ***P < 0.001.

## Conflict of interest

IW's laboratory has received funding from CSL Innovation Ltd for research on antagonists of haemopoietic growth factors, including G‐CSF. CG, RB, TYT, MA and NW have been or were employed by CSL Innovation at the time of manuscript preparation.

## Author contributions

**Katherine R Martin:** Data curation; formal analysis; funding acquisition; investigation; methodology; project administration; supervision; validation; visualization; writing – original draft; writing – review and editing. **Cristina Gamell:** Formal analysis; investigation; methodology; resources; validation; visualization; writing – original draft; writing – review and editing. **Tsin Yee Tai:** Data curation; formal analysis; investigation; methodology; validation. **Roberto Bonelli:** Formal analysis; investigation; methodology; resources; validation. **Jacinta Hansen:** Investigation. **James Tatoulis:** Investigation; methodology; resources; writing – review and editing. **Monther Alhamdoosh:** Methodology; resources; software; validation. **Nicholas Wilson:** Conceptualization; methodology; project administration; resources; supervision; writing – original draft; writing – review and editing. **Ian Wicks:** Conceptualization; funding acquisition; methodology; project administration; resources; supervision; writing – original draft; writing – review and editing.

## Supporting information


Supplementary tables 1–3

## Data Availability

Whole cell transcriptomics data have been deposited into the NCBI GEO database, with the dataset identifier GSE253782.
