# Supplementary material for: Whole blood transcriptomics reveals granulocyte colony‐stimulating factor as a mediator of cardiopulmonary bypass‐induced systemic inflammatory response syndrome
Source: Clin Transl Immunology. 2024 Feb 19;13(2):e1490. doi: 10.1002/cti2.1490 (PMC10875393; doi:10.1002/cti2.1490)
Supplement: Supplementary file 1 — Supplementary tables 1–3 [file CTI2-13-e1490-s001.docx]

**Supplementary table 1. Clinical outcomes definitions**

| **Outcome** | **﻿Description** |
| --- | --- |
| New renal Insufficiency | ﻿Acute post-operative renal insufficiency characterised by one of the following:   1. Increased serum creatinine to > 0.2 mmol/L (> 200 µmol/L) and a doubling or greater increase in creatinine over the baseline pre-operative value and the patient did not require preoperative dialysis/hemofiltration. 2. A new post-operative requirement for dialysis/hemofiltration when the patient did not require this pre-operatively. |
| Peri/Post-operative myocardial infarction (MI) | ﻿Peri-/post- operative MI diagnosed by finding at least two of the following criteria:   1. Enzyme level elevation either:  - CK-MB > 30 units OR - Troponin > 20.0 micrograms/L OR - Troponin level equivalent documented at your instruction, provided operation does not involve myocardial incision.  1. new wall motion abnormalities 2. At least two serial ECG showing Q waves, duration ≥ 0.03ms in 2 contiguous leads |
| Peri/Post-operative cardiogenic shock | ﻿Peri-/post- operative cardiogenic shock fulfilled all of the following criteria:   1. Sustained (>30 minutes) episode of systolic blood pressure < 90 mm Hg or the requirement for parenteral inotropic or vasopressor agents or mechanical support (e.g., Intra-aortic balloon pump (IABP), extracorporeal circulation, ventricular assist devices to maintain BP > 90 mm Hg) 2. Evidence of elevated filling pressures (e.g., pulmonary congestion on examination or chest radiograph) 3. Evidence of end organ hypoperfusion (e.g. urine output 30 mL/h, or cold/diaphoretic extremities, or obtunded mental status if previously normal, etc.) |
| ﻿Cardiac arrest | ﻿Patient had a new cardiac arrest diagnosed by one or more of the following:   1. Ventricular fibrillation 2. Rapid ventricular tachycardia with haemodynamic instability 3. Asystole 4. Pulseless electrical activity (PEA) |
| Stroke | Patient experienced a stroke or new central neurologic deficits persisting for > 72 h peri- or post-operatively. Neurological deficits were characterised by persistent loss of neurological function caused by an ischaemic or haemorrhagic event. |
| Pulmonary embolism | ﻿Pulmonary embolism diagnosed by studies such as ventilation/perfusion (V/Q) scan or angiogram |
| ﻿Pneumonia | Diagnosed post-operatively by one of the following:   1. Positive cultures of sputum or trans-tracheal aspirate 2. Clinical, including haematological findings consistent with the diagnosis of pneumonia and radiographic evidence |
| Wound infection | Wound infections include:   1. Deep sternal wound infections 2. Superficial access-wound infections 3. Donor site deep wound infections 4. Deep access wound infection of parasternal site |
| ﻿Septicaemia | ﻿Defined by positive blood cultures supported by at least two of the following indices of clinical infection:   1. Fever 2. Elevated granulocyte cell counts. 3. Elevated and increasing C-reactive protein (CRP) 4. Elevated and increasing erythrocyte sedimentation rate (ESR) post-operatively. |
| ﻿Aortic dissection | Dissection in any part of the aorta |
| ﻿Anticoagulant complications | ﻿Patient developed bleeding, haemorrhage, and/or embolic events related to anticoagulant therapy |
| ﻿Gastrointestinal tract complications | GIT complication including any of the following:   1. GI bleeding requiring transfusion. 2. Pancreatitis with abnormal amylase/lipase requiring nasogastric suction therapy. 3. Cholecystitis requiring cholecystectomy or drainage. 4. Mesenteric ischemia requiring exploration. 5. Hepatitis 6. Other GI complication |
| ﻿Multi-system failure | ﻿For this diagnosis to be made, two or more of the following major organ systems must fail concurrently for at least 48 h:   1. Renal – new renal failure 2. Respiratory – requires endotracheal intubation for respiratory dysfunction. 3. Cardiac – the use of inotropes and/or IABP to treat low cardiac output. 4. Hepatic failure on the basis of enzymes, and bilirubin estimation |

**Supplementary table 2. Summary of detected DE genes.**

| **Contrast** | **Down** | **Up** | **NotSig** | **FDR.Cutoff** | **FC.Cutoff** |
| --- | --- | --- | --- | --- | --- |
| X60 - PRE | 3742 | 2801 | 5475 | 0.05 | 1 |
| Day_1 - PRE | 3852 | 3591 | 4575 | 0.05 | 1 |
| Day_1 - X60 | 3861 | 3798 | 4359 | 0.05 | 1 |
| X60 - PRE | 726 | 867 | 10425 | 0.05 | 1.5 |
| Day_1 - PRE | 1678 | 1350 | 8990 | 0.05 | 1.5 |
| Day_1 - X60 | 1451 | 1307 | 9260 | 0.05 | 1.5 |
| X60 - PRE | 124 | 369 | 11525 | 0.05 | 2 |
| Day_1 - PRE | 585 | 436 | 10997 | 0.05 | 2 |
| Day_1 - X60 | 550 | 529 | 10939 | 0.05 | 2 |
| X60 - PRE | 4180 | 3086 | 4752 | 0.1 | 1 |
| Day_1 - PRE | 4179 | 3872 | 3967 | 0.1 | 1 |
| Day_1 - X60 | 4213 | 4114 | 3691 | 0.1 | 1 |
| X60 - PRE | 728 | 871 | 10419 | 0.1 | 1.5 |
| Day_1 - PRE | 1686 | 1354 | 8978 | 0.1 | 1.5 |
| Day_1 - X60 | 1453 | 1310 | 9255 | 0.1 | 1.5 |
| X60 - PRE | 124 | 369 | 11525 | 0.1 | 2 |
| Day_1 - PRE | 585 | 438 | 10995 | 0.1 | 2 |
| Day_1 - X60 | 550 | 529 | 10939 | 0.1 | 2 |
| X60 - PRE | 4473 | 3309 | 4236 | 0.15 | 1 |
| Day_1 - PRE | 4423 | 4137 | 3458 | 0.15 | 1 |
| Day_1 - X60 | 4416 | 4314 | 3288 | 0.15 | 1 |
| X60 - PRE | 729 | 872 | 10417 | 0.15 | 1.5 |
| Day_1 - PRE | 1688 | 1360 | 8970 | 0.15 | 1.5 |
| Day_1 - X60 | 1454 | 1310 | 9254 | 0.15 | 1.5 |
| X60 - PRE | 124 | 370 | 11524 | 0.15 | 2 |
| Day_1 - PRE | 585 | 441 | 10992 | 0.15 | 2 |
| Day_1 - X60 | 550 | 529 | 10939 | 0.15 | 2 |

**Supplementary table 3. List of primers**

| Gene | Forward Primer | Reverse Primer |
| --- | --- | --- |
| *COX2* | CTCCCGATTGAAGCCCCCATT | GGCATGAAACTGTGGTTTGCTCC |
| *ND1* | ACCTCCTACTCCTCATTGTACC | GTTTTATGGCGTCAGCGAAG |
| *D-Loop* | ATCCCGCACAAGAGTGCTAC | GGGGAACGTGTGGGCTATTT |
